# Genetically Predicted 3-Methoxytyrosine Mediates the Causal Association between Fibroblast Growth Factor 21 and Glioblastoma Multiforme

**DOI:** 10.7150/jca.98035

**Published:** 2025-01-01

**Authors:** Xuan Chen, Lihui Han, Wenzhe Xu

**Affiliations:** 1 Department of Radiation Oncology, Qilu Hospital of Shandong University, West Wenhua Rd. 107, Jinan 250012, China.; 2 Department of Neurosurgery, Qilu Hospital of Shandong University and Institute of Brain and Brain-Inspired Science, Shandong University, West Wenhua Rd. 107, Jinan 250012, China.; 3 Shandong Key Laboratory of Brain Function Remodeling, West Wenhua Rd. 107, Jinan 250012, China.

**Keywords:** glioblastoma multiforme, fibroblast growth factor 21, 3-methoxytyrosine, mendelian randomization, mediation analysis

## Abstract

**Background:** Glioblastoma multiforme (GBM) is one of the most common brain malignancies characterized by an inflammatory microenvironment and metabolic reprogramming. This study aims to investigate the causal relationship between inflammatory factors (IFs) and GBM, as well as the potential mediating effects of specific plasma metabolites.

**Methods:** We used a bidirectional two-sample Mendelian randomization (MR) approach to investigate the causal associations between 91 IFs and GBM. We employed a two-step MR technique to identify significant mediators in this relationship, followed by a mediation analysis to explore and quantify the mediating effects of specific metabolites on the causal relationship between IFs and GBM. *In vitro* experiments were conducted to verify the effects of specific IF and metabolite on GBM cells. The response of cells to treatment was examined using a series of assays, including colony formation, cell proliferation, and migration assays.

**Results:** Three IFs showed significant associations with GBM. Among them, fibroblast growth factor 21 (FGF21) had a protective effect against GBM [odds ratio (OR): 0.42; 95% confidence interval (CI): 0.25, 0.71; p=1.00×10^-3^]. There was no strong evidence that genetically predicted GBM had an effect on FGF21 (OR: 1.04; 95% CI: 0.83, 1.31; p = 0.692). Mediation analysis identified 3-methoxytyrosine (3-MTyr) level (mediation effect of 11.50%) as a significant intermediary. The *in vitro* study demonstrated that FGF21 inhibited proliferation and migration in GBM cells, whereas 3-MTyr exerted the opposite effects.

**Conclusion:** FGF21 was causally associated with a reduced risk of GBM, and this relationship is partially mediated by 3-MTyr. This identified regulatory network offers a novel avenue for further research into the pathogenic mechanisms of GBM and provides a theoretical foundation for the development of relevant therapeutic regimens.

## Introduction

Glioblastoma multiforme (GBM) is the most common and aggressive primary brain tumor in adults. It has a poor response to the aggressive multimodal treatment, including surgery, radiotherapy, and chemotherapy 1. The major obstacles for the development of efficient treatments are a high degree of heterogeneity, angiogenesis, invasiveness, and the presence of tumor stem cells that are resistant to chemoradiotherapy 2. Identifying new driving factors for GBM progression could provide a deeper understanding of the disease and serve as predictors for prognosis and targets for precise treatment.

Surgically removed GBM tissue often exhibits local brain inflammation, increased levels of inflammatory factors (IFs), and activation of inflammatory signaling pathways 3, 4. These inflammatory cells and mediators could contribute to GBM progression and the molecular evolution of GBM cells 5, 6. As a result, targeting transcription factors or kinases related to inflammation has become a promising therapy for GBM. In this tumor microenvironment, GBM cells undergo metabolic reprogramming, including changes in the metabolism of the amino acids glutamate, tryptophan, and arginine 6. Plasma metabolite profiles are a promising method for identifying new biomarkers for GBM, including early detection, diagnosis, prognosis, and drug efficacy assessment 7-9. However, it is unclear whether plasma metabolites have a causal effect on GBM risk, and it is unknown if inflammatory factors (IFs) contribute to metabolic alterations.

Mendelian randomization (MR) is a powerful epidemiological research method that essentially uses genetic variation as instrumental variables (IVs) to identify causal relationships between risk factors and disease outcomes 10. We hypothesized that the effect of inflammation on GBM development might be mediated through specific blood metabolites, given the underexplored metabolic mechanism of inflammation in GBM progression and the important role of metabolic reprogramming in GBM pathogenesis. We conducted a two-sample, two-step MR study to investigate the causal role of plasma metabolites in linking the effect of IFs on GBM. Our data showed a negative correlation between IF fibroblast growth factor 21 (FGF21) and the risk of GBM. We also identified metabolite 3-methoxytyrosine (3-MTyr) as a significant mediator in this relationship.

## Materials and Methods

### Study design

**Figure 1:**
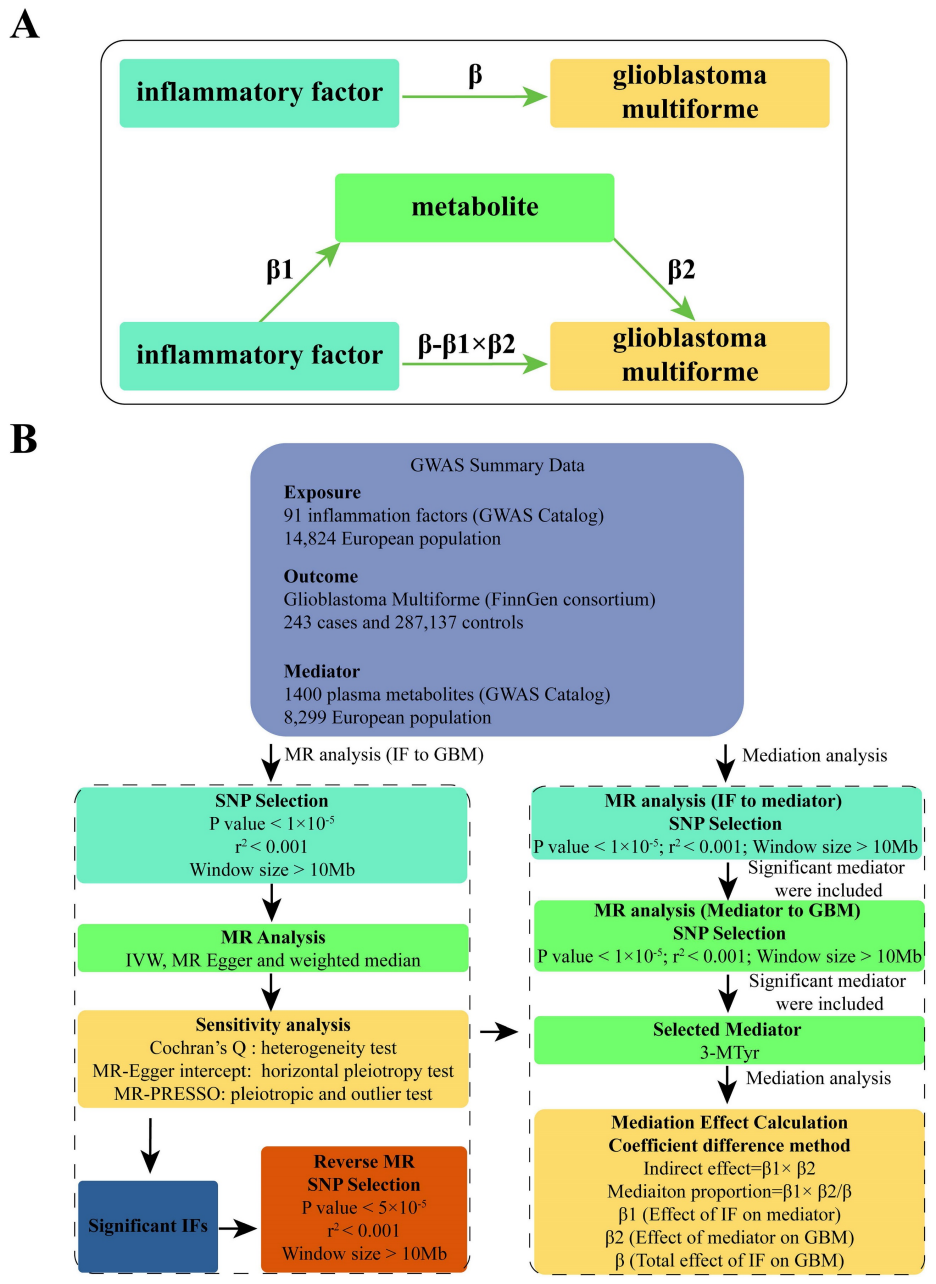
(A) Diagrams illustrating the associations examined in this study are presented. The total effect (β) between IF and GBM was decomposed into two parts: (i) the indirect effect, which was calculated using a two-step approach (where β1 represents the effect of IF on metabolite, and β2 represents the effect of metabolite on GBM) and the coefficient difference method (β1×β2); and (ii) the direct effect (β-β1×β2). (B) A flow chart outlining the methodology applied in our study is provided. SNP selection criteria were applied for MR analysis to determine causal relationships and identify significant IF. Mediation analysis quantified the potential influence of 3-MTyr on the IF-GBM association. Abbreviations: GWAS, genome-wide association study; MR, mendelian randomization; IF, inflammatory factor; GBM, glioblastoma multiforme; SNP, single nucleotide polymorphism; IVW, inverse variance weighted; MR-PRESSO, MR-Pleiotropy Residual Sum and Outlier, 3-MTyr, 3-methoxytyrosine.

We used a two-sample MR approach to examine the causal relationship between 91 IFs and GBM. This method employed genetic variants, specifically single nucleotide polymorphisms (SNPs), as IVs. To ensure the reliability and directionality of our results, we conducted sensitivity analyses and reverse MR. Given the complex interplay between inflammation and metabolism, we selected 1400 plasma metabolites as potential mediators. A two-step approach using MR was implemented to identify significant mediators. 11. Subsequently, mediation analysis was utilized to estimate the direct effect of IF on GBM, adjusting for the significant mediators. 12. This allowed for the calculation of the proportion of the effect of IFs on GBM that is mediated through specific metabolites. Please refer to Figure 1 for a flow chart illustrating our study design and analytical steps.

### Data source

The GWAS Catalog was used to obtain summary statistics for inflammation-related plasma protein (n=14,824) (Accession numbers GCST90274758-90274848) and plasma metabolites (n=8,299) (Accession number: GCST90199621-90201020). The FinnGen consortium R9 release data (243 cases and 287,137 controls) (https://r9.finngen.fi/) was used to obtain the GBM data.

### The selection of IVs

We selected potential IVs from the genome-wide association study (GWAS) summary data for each exposure based on SNPs that showed a genome-wide significant association (p<5.0×10^-8^). Due to a limited number of available IVs, we adjusted the significance threshold to p<1.0×10^-5^ for both IFs and metabolites. For the reverse MR analysis involving GBM, we set the significance threshold to p<5.0×10^-5^ to require more than 20 SNPs. Afterwards, SNPs were grouped together to account for the effect of linkage disequilibrium. This was done by excluding SNPs with an r^2^ value less than 0.001 and a window size greater than 10Mb. The harmonization process removed palindromic and incompatible alleles. To avoid weak instrument bias, SNPs with a minor F-statistic less than 10 were eliminated. The F-statistic was calculated using the following equation: F = R^2^ * (n - 2) / (1 - R^2^), where R^2^ and n represent the exposure variance of the IVs and the sample size, respectively.

### MR analysis

We used a bidirectional two-sample MR analysis to investigate the causal relationship between 91 IFs and GBM. Multiple analytical approaches were applied, including inverse variance weighted (IVW), MR Egger, and weighted median. When horizontal pleiotropy is not present, we used the IVW test as the main method to calculate unbiased estimates of causal effects. The other two methods were considered supplementary to the IVW approach. We selected the most significant IF for further study. Then sensitivity analysis and reverse MR were conducted to confirm the absence of horizontal pleiotropy and reverse causality. For reverse MR, the significance threshold for selecting IVs was set at P<5.0×10-5. All other methods and settings were consistent with those used in forward MR.

To select blood metabolites, we conducted MR analysis to assess the causal relationship between the chosen IF and 1400 metabolites. We then included the significant metabolites as the exposure and used GBM as the outcome for the subsequent MR analysis. Finally, we selected the most significant metabolite as the mediator for the mediation MR analysis.

We conducted a mediation analysis using a two-step MR design to investigate whether plasma metabolites mediate the causal pathway from IF to GBM outcome (Figure 1B). We used the IVW approach to determine the total effect of IF on GBM (β), the effect of IF on metabolites (β1), and the effect of metabolites on GBM risk (β2). To calculate the indirect mediation effect of metabolites on GBM outcome, we used the coefficient difference method, which involves calculating the causal effect of IF on GBM via metabolites (β1 × β2) 13. The direct effect was estimated by adjusting for the mediator (β - β1 × β2). We calculated the percentage mediated by dividing the indirect effect by the total effect (β1 × β2/β) (Figure 1A).

### Sensitivity analysis

The IVW approach utilized Cochran's Q test to assess heterogeneity resulting from diverse SNPs. A P-value above 0.05 suggests non-heterogeneity. To detect potential horizontal pleiotropy, the MR-Egger intercept test was conducted. An intercept P-value < 0.05 indicates a significant pleiotropic bias. The MR-Pleiotropy Residual Sum and Outlier (MR-PRESSO) method was used to validate the results of the IVW model and correct for any outliers. If any outliers were present, they were removed, and the analysis was repeated. The stability of the causal estimates was evaluated through a leave-one-out analysis. Each SNP was sequentially excluded to assess if any single SNP had a potential impact on the overall results. The TwoSampleMR package (version 0.5.7) in R software (version 4.2.1) was used for all analyses. The study considered p < 0.05 to be statistically significant.

### Cell culture

The human GBM cell lines U118 and U251 were procured from the Chinese Academy of Sciences Cell Bank and maintained in DMEM supplemented with 10% fetal bovine serum (FBS) (Gibco, Waltham, MA, USA). The cell lines were authenticated by the Genetic Testing Biotechnology Corporation (Suzhou, China) using short tandem repeat markers and confirmed to be free of mycoplasma contamination using the Myco-Lumi™ Luminescent Mycoplasma Detection Kit (Beyotime, Nantong, China). The cells were incubated with 50ng/ml of FGF21 (Medchemexpress, Shanghai, China) for 24 hours or 500 μmol/L of 3-MTyr for 30 minutes. The concentration was selected based on the findings of previous studies 14, 15.

### Colony formation assay

The GBM cells were seeded into 6-well plates at a density of 1000 cells per well. After treatment, cells were incubated for a further 10 days. Subsequently, the colonies were fixed with ethanol and stained with crystal violet. Colonies comprising a minimum of 50 cells were deemed to be surviving colonies. The survival curves were plotted using Prism 7.0 (GraphPad Inc., La Jolla, CA, USA).

### 5-Ethynyl-2´-deoxyuridine (EdU) proliferation assay

GBM cells were plated on coverslips and stained with EdU using an EdU incorporation assay kit (Ribobio, Guangzhou, China) according to the manufacturer's protocol. The nuclei were counterstained with DAPI and the Edu-positive cells were visualized under fluorescence microscopy (Leica DMi8; Wetzlar, Germany). Five fields on each coverslip were randomly imaged, and the experiment was repeated three times. The number of EdU/DAPI-positive cells were enumerated using Image-pro plus 6.0 (Media Cybernetics Inc., Silver Spring, MD, USA) and the mean per coverslip was calculated.

### Cell migration assay

Cell migration assay was conducted using the Transwell system (8 μm pore, Corning Costar, Corning, NY, USA). A suspension of cells at a density of 5×10^5^/ml in serum-free medium were seeded in the upper chamber, while the lower chamber was filled with medium containing 10% FBS. Following a 24-hour incubation period, the cells remaining on the upper surface were removed with cotton swabs, while the cells that had migrated into the lower surface were fixed, stained with crystal violet and then counted.

### Statistical analysis of the *in vitro* experiments

The data were presented as the mean ± standard deviation (SD) and analyzed with Prism 7.0. Statistical analyses were performed using one-way analysis of variance with the post hoc Tukey's test. P<0.05 was considered statistically significant.

## Results

### MR analysis of IFs' effect on GBM

**Figure 2:**
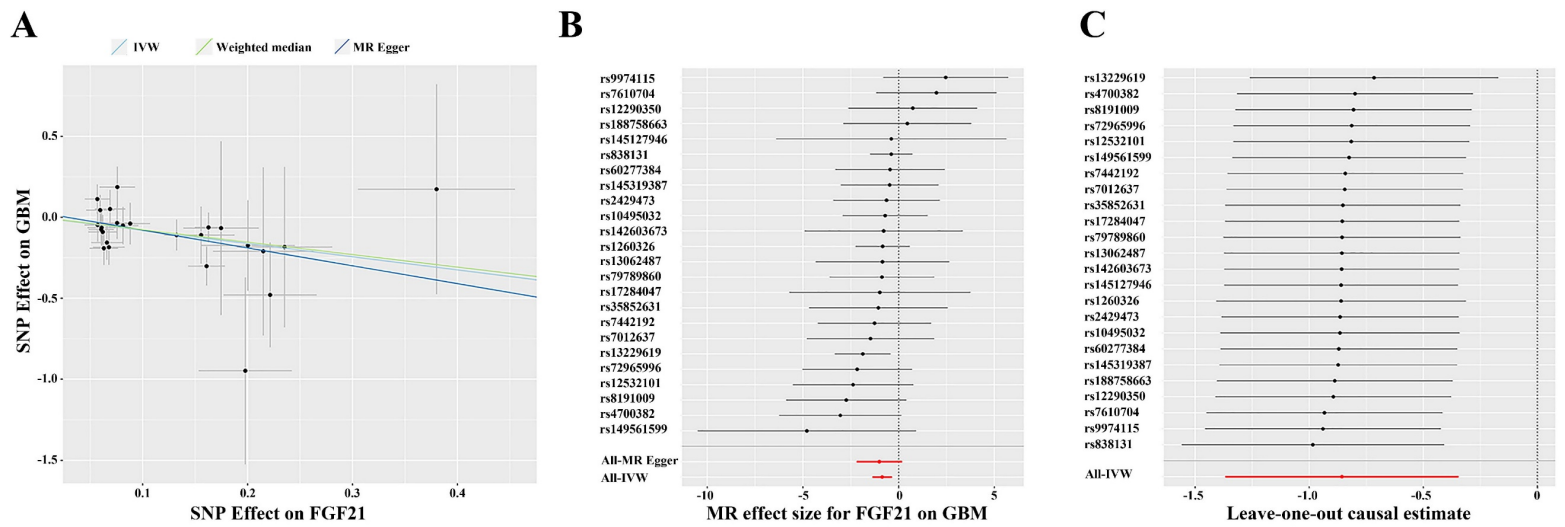
(A) Scatter plot for the causal association between FGF21 and GBM. (B) Forest plot of the causal effects of SNPs associated with FGF21 on GBM. Red lines represent estimations from the MR Egger and IVW test. (C) Leave-one-out plots for the causal association between FGF21 and GBM. Red lines represent estimations from the IVW test. Abbreviations: IVW, inverse variance weighted; MR, mendelian randomization; SNP, single nucleotide polymorphism; GBM, glioblastoma multiforme; FGF21, fibroblast growth factor 21.

**Figure 3:**
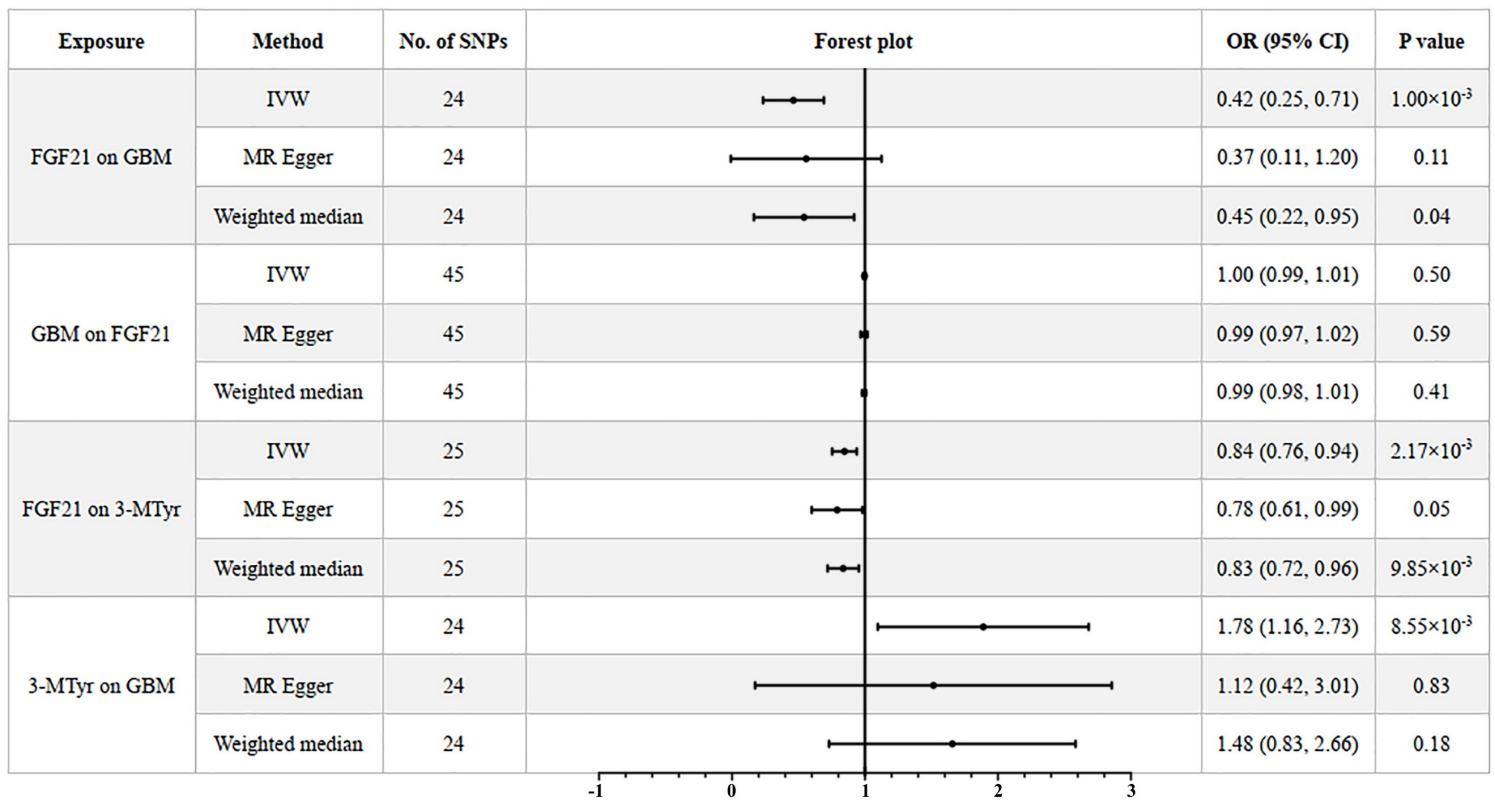
Forest plot to visualize the causal effects of 3-MTyr with FGF21 and GBM. Three methods: IVW, MR Egger and weighted median. Abbreviations: FGF21, fibroblast growth factor 21; GBM, glioblastoma multiforme; 3-MTyr, 3-methoxytyrosine; IVW, inverse variance weighted; OR, odds ratio; CI, confidence interval.

After analyzing the associations between 91 IFs and GBM risk, we found suggestive evidence that 3 IFs were associated with GBM using the IVW method (p=0.04 for Fractalkine levels; p=0.03 for C-X-C motif chemokine 6 levels and p=1.00×10-3 for FGF21). FGF21 had the highest significance [odds ratio (OR): 0.42; 95% confidence interval (CI): 0.25, 0.71; p=1.00×10-3] (Figure 2B and 3). The results obtained from the Weighted Median methodology (OR: 0.45; 95% CI: 0.22, 0.95; p=0.04) were consistent with the findings obtained from the IVW method (Figure 2A). The Cochran's Q test indicated no significant heterogeneity among these IVs (p=0.75). Additionally, the MR-PRESSO test did not detect any outliers (Global Test p=0.76). The leave-one-out analysis results further confirmed the absence of any potential outliers (Figure 2C). The MR-Egger regression intercept analysis did not reveal any potential directional horizontal pleiotropy (p=0.79). In reverse MR analysis, we found no significant association between GBM and FGF21 (OR: 1.00; 95% CI: 0.99, 1.01; p=0.50) (Figure 3).

### Mediation analysis of FGF21 on GBM

In our investigation to clarify the role of FGF21 in GBM through plasma metabolites, we used a two-step MR approach. Using the IVW approach, we found that FGF21 was associated with 203 metabolites. Among these, the IVW estimate suggested that 20 metabolites were associated with GBM. We eliminated 4 metabolites without detailed information and selected the most significant metabolite, 3-MTyr, for mediation analysis.

The genetically predicted FGF21 showed a negative correlation with the level of 3-MTyr (OR: 0.84; 95% CI: 0.76, 0.94; p=2.17×10^-3^). The weighted median method produced consistent results (OR: 0.83; 95% CI: 0.72, 0.96; p=9.85×10^-3^). In turn, elevated levels of 3-MTyr were positively associated with an increased risk of GBM (OR: 1.78; 95% CI: 1.16, 2.73; p=8.55×10^-3^) (Figure 3).

After conducting mediation analysis, we calculated that FGF21 has a significant indirect effect on GBM through 3-MTyr levels, accounting for approximately 11.50% of the total effect. This was evidenced by an indirect effect (β1× β2) of -0.099 and a total effect (β) of -0.857.

### FGF21 resulted in a notable decline in proliferation, colony formation and migration in GBM cells *in vitro*, whereas 3-MTyr elicited the opposite effects

**Figure 4:**
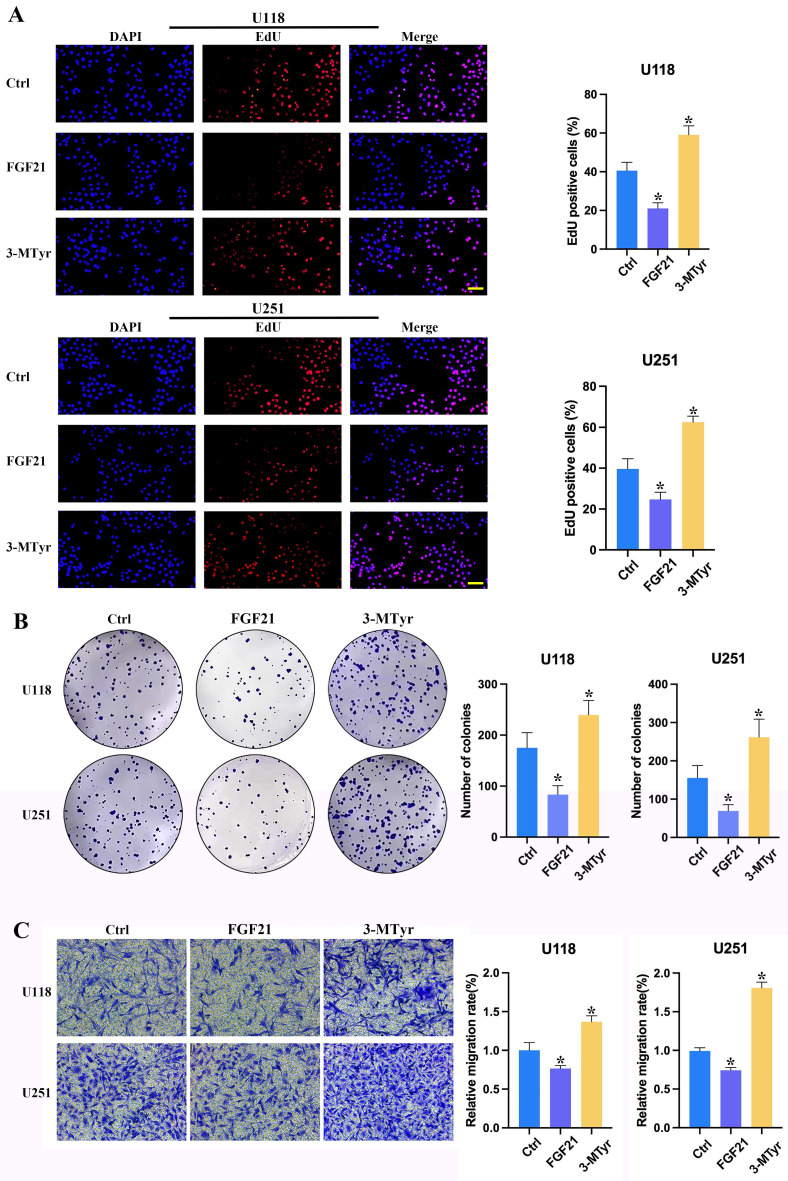
(A) EdU proliferation assay of U118 and U251 cells. Cells were labeled with Apollo 567 and the nuclei were counterstained with DAPI. Scale bar = 200 μm. (B) Colony formation of U118 and U251 cells after treatment with FGF21 or 3-MTyr. (C) Transwell assay of U118 and U251 cells. Data are presented as the mean ± SD (n=3). *p < 0.05. Abbreviations: EdU, 5-Ethynyl-2´-deoxyuridine; FGF21, fibroblast growth factor 21; 3-MTyr, 3-methoxytyrosine.

To ascertain the impact of FGF21 and 3-MTyr on GBM cells, U118 and U251 cells were treated with FGF21 or 3-MTyr, and the subsequent effects on proliferation, colony formation and migration were evaluated. The impact of FGF21 and 3-MTyr on cell proliferation was assessed through the incorporation of EdU. The data revealed that Edu-positive cells exhibited a decline in response to FGF21, whereas an increase was observed following 3-MTyr treatment (Figure 4A). These findings were corroborated by the colony formation assay (Figure 4B). Subsequently, the effect of FGF21 on cell migration was investigated. Following treatment with FGF21, the number of migrating cells increased, while 3-MTyr had the opposite effect (Figure 4C).

## Discussion

The FGF21 was defined as a neural gene 16, but its causal relation with GBM remains elusive. Our study aimed to determine the causal effects between FGF21 and GBM. We used MR analysis to investigate the association between FGF21 and GBM based on existing GWAS and to explore the mediating effects of specific metabolites. Our results suggested that genetically predicted FGF21 was associated with a decreased risk of GBM, and 11.50% of this effect was mediated through 3-MTyr.

We are the first to investigate the causal relationship between FGF21 and the risk of GBM through MR methods, while also demonstrating 3-MTyr as their mediator. FGF21 is a growth factor that has been reported to protect against various insults, such as cardiac hypertrophy, sepsis toxicity, diabetes-induced cardiac cell apoptosis, high glucose-induced endothelial cell damage, and glutamate-induced neuron death 17-21. FGF21 is a potent metabolic regulator, rather than a growth promoter. The transgenic mouse model demonstrated that FGF21 inhibited the development of chemically-induced hepatic tumors, which contradicts the common assumption that growth factors promote cell growth 22. Additionally, FGF21 deficiency was associated with an increased risk of prostate cancer, clear cell renal cell carcinoma and breast cancer 23. In this study, we found a negative association between FGF21 and GBM risk, suggesting a protective role for FGF21 in central nervous system (CNS) pathological conditions.

Previous studies have shown that FGF21 plays a role in CNS or between the CNS and systemic organs. For example, when rodents were given intracerebroventricular injections of FGF21, their hepatic insulin sensitivity and metabolic rate increased in cases of diet-induced obesity 24. Moreover, a decrease in FGF21 levels was identified in the serum of individuals diagnosed with Parkinson's disease and Alzheimer's disease. The administration of FGF21 has been demonstrated to mitigate the associated neural pathologies and degeneration 25. FGF21 is capable of crossing the blood-brain barrier and directly reaching the brain, where it interacts with its receptor β-Klotho, which is selectively expressed 26. The interaction between FGF21 and β-Klotho regulates neuron function, extends glial cell process outgrowth, and attenuates neuroinflammation and oxidative stress 27, 28. Hypoxia is the hallmark of the GBM microenvironment, which promotes malignant progression, growth, and therapeutic resistance by inducing oxidative stress and inflammation. The antioxidant and anti-inflammatory capacity of FGF21 may contribute to the negative relationship between FGF21 and GBM. However, the detailed mechanism requires further investigation. Moreover, pathway and functional enrichment analyses showed that FGF21 exerts its influence on GBM through glucose response mechanisms 29. The metabolic alterations that are characteristic of GBM may be inhibited by the regulation of the abnormal metabolic state through FGF21, which could potentially lead to a reduction in GBM progression.

As FGF21 is a metabolic messenger 30, the altered metabolic pathways may contribute to the effects of FGF21 on GBM. Our study utilized the latest metabolites GWAS resource and found the potential mediating role of 3-MTyr. Metabolite 3-MTyr is considered as an indicator of dopamine catabolism 31. Alterations in 3-MTyr have also been demonstrated in bladder cancer and melanoma 32, 33 Furthermore, 3-MTyr could be regarded as a promising novel diagnostic and prognostic biomarker for neuroblastoma 34, 35. In this study, we demonstrated that 3-MTyr mediated the negative causal effects of FGF21 on GBM. The role of 3-MTyr in cancer progression remains unclear, and there is limited research on its association with neoplasia. Therefore, our study offers a novel perspective for bio-functional and mechanistic research on this metabolite. Previous studies have shown that FGF21 increases dopamine levels 36, 37, which in turn reduces the content of 3-MTyr by decreasing dopamine degradation. These findings support our conclusion that FGF21 is negatively correlated with 3-MTyr.

The proportion of 3-MTyr mediated was 11.50%. Our mediation analysis also suggested that FGF21 may decrease the risk of GBM through other important mediators, such as glycerophosphoethanolamine (GPE), a byproduct of phospholipid metabolism. GPE was found to be decreased in gadolinium contrast-enhancing/necrotic regions of GBM. Additionally, differences in GPE could discriminate between GBM and normal brain tissue 38. It has been proposed that the reduction of GPE favors the downregulation of phospholipid metabolism, possibly as a means for the cell to limit the release of membrane-originating fatty acids to the cytosol and the propagation of oxidative stress 39. Therefore, we hypothesize that the metabolic alteration mediated by FGF21 in GBM leads to decreased membrane turnover in order to restrain cell proliferation 38. However, the detailed mechanism still requires investigation, and further studies are necessary to quantify other mediators.

Our analyses have led to the construction of a regulatory network encompassing IFs, metabolites and GBM, which provides a novel insight into the role of abnormal metabolism in GBM pathogenesis. Our study offers crucial perspectives into how FGF21 may affect the risk of GBM by regulating specific metabolites. FGF21 is a metabolic regulator and is primarily secreted by liver cells 29. Furthermore, FGF21 has been identified as a potential modulator in liver-mediated inter-organ crosstalk. Hepatic FGF21 has been demonstrated to regulate glucocorticoid production in CNS, thereby establishing an important liver-brain axis that can be employed to modulate the brain-regulated adaptive response 25. Previous study has found that liver disease could lead to dementia through neuroinflammation 40. It can thus be hypothesized that FGF21 may affect the occurrence of GBM via the liver-brain axis. This provides a promising direction for future research into the influence of the liver-brain axis on GBM. Increased FGF21 levels were observed in patients with liver tumor, non-small cell lung cancer, breast cancer, clear renal cell carcinoma and endometrioid carcinoma. In papillary thyroid carcinoma, there was a positive correlation between FGF21 levels and histological grade, recurrence, and mortality 25. It is therefore recommended that any aberrant FGF21 levels detected in neuronal diseases in both the brain and blood be identified with caution. Furthermore, future research should focus on the application of FGF21 in assessing the prognosis and therapeutic efficacy in GBM. The identified regulatory network of the live-brain axis may play a pivotal role in GBM progression, offering invaluable insights for future research and therapeutic interventions.

Our study has several strengths. Firstly, we are the first to explore the causal effects of FGF21 on the risk of GBM using MR analysis. Secondly, the use of MR analysis reduces bias caused by confounders and potential reverse causality compared to conventional observational studies. Therefore, our analysis provides more convincing evidence to support the causality of FGF21 and GBM. Multiple sensitivity analyses could ensure the statistical power of our study's findings and conclusions. Additionally, our study's findings are novel. The metabolic pathway of 3-MTyr in mediating the inhibitory effect of FGF21 on GBM could help in understanding the role of metabolic reprogramming and offer new directions for future research. However, this study has several limitations. The generalizability of our findings to other ethnic populations is limited due to the majority of participants enrolled in these GWAS being of European descent. Although numerous sensitivity analyses were performed, the potential impact of unmeasured confounding factors on our findings cannot be entirely dismissed. IVs with a threshold of p<1×10-5 were chosen to ensure adequate IVs, surpassing the traditional GWAS significance threshold of p<5×10-8. In future studies, we aim to expand the sample size to investigate the correlation between IF and GBM more thoroughly. Additionally, we used summary-level statistics in our study, rather than individual-level data, which limits our ability to explore causal links between subgroups such as females and males. Further stratified analyses would enhance our understanding of the association between FGF21 and GBM.

## Conclusions

Our MR analysis revealed a causal relationship between FGF21 and GBM, with 11.50% of the effect mediated by 3-MTyr. These findings highlight the significant role of IFs in metabolic reprogramming and provide a new perspective on the potential determinants of GBM risk. We anticipate that these insights will lead to the development of innovative strategies that could significantly impact the long-term management and prognosis of GBM.

## Abbreviations

GBM: glioblastoma multiforme
IF: inflammatory factor
MR: mendelian randomization
FGF21: fibroblast growth factor 21
OR: odds ratio
CI: confidence interval
3-MTyr: 3-methoxytyrosine
IV: instrumental variable
SNP: single nucleotide polymorphism
GWAS: genome-wide association study
IVW: inverse variance weighted
MR-PRESSO: MR-Pleiotropy Residual Sum and Outlier
FBS: fetal bovine serum
EdU: 5-Ethynyl-2´-deoxyuridine
SD: standard deviation
CNS: central nervous system
GPE: glycerophosphoethanolamine
